# Onset of the Mach Reflection of Zel’dovich–von Neumann–Döring Detonations

**DOI:** 10.3390/e23030314

**Published:** 2021-03-06

**Authors:** Tianyu Jing, Huilan Ren, Jian Li

**Affiliations:** State Key Laboratory of Explosion Science and Technology, Beijing Institute of Technology, Beijing 100081, China; 3120170078@bit.edu.cn (T.J.); huilanren@bit.edu.cn (H.R.)

**Keywords:** ZND detonation, mach reflection, frozen-limit, length scale, numerical simulation

## Abstract

The present study investigates the similarity problem associated with the onset of the Mach reflection of Zel’dovich–von Neumann–Döring (ZND) detonations in the near field. The results reveal that the self-similarity in the frozen-limit regime is strictly valid only within a small scale, i.e., of the order of the induction length. The Mach reflection becomes non-self-similar during the transition of the Mach stem from “frozen” to “reactive” by coupling with the reaction zone. The triple-point trajectory first rises from the self-similar result due to compressive waves generated by the “hot spot”, and then decays after establishment of the reactive Mach stem. It is also found, by removing the restriction, that the frozen limit can be extended to a much larger distance than expected. The obtained results elucidate the physical origin of the onset of Mach reflection with chemical reactions, which has previously been observed in both experiments and numerical simulations.

## 1. Introduction

The Mach reflection of detonation waves is a classical problem that attracts considerable research attention, even in problems with multi-interfaces [1,2,3]. Because of the complexity of the Mach reflection of detonations, which involve hydrodynamics, chemical release, and length scale effects, the fundamental physics remains unclear and is prone to contradictions and misunderstandings. This most likely results from the widespread application of the classical three-shock theory for shocks [4,5,6] and the reactive three-shock theory for detonation discontinuities (by ignoring the thickness) [7,8,9,10,11], both of which assume self-similarity and a pseudo-steady state. However, even though a few investigators have reported the absence of self-similarity based on experimental observations or numerical simulations [8,10,12,13,14], three-shock theory has nevertheless been applied to compare with the results. Obviously no general agreement has been obtained [15,16,17]. Following the work of Hornung et al. [18] and Sandeman et al. [15], who both studied the Mach reflection phenomena of dissociating strong shock waves, Akbar [8] and Shepherd et al. [10] first turned research attention to the failure of self-similarity due to characteristic length scales (i.e., cell size λ, reaction length Δ, or hydrodynamic length *H*), and introduced the concept of frozen and equilibrium limits to the Mach reflection of detonations on a wedge. The frozen limit applies when the Mach stem travels a sufficiently small distance as compared to λ. In contrast, when the Mach stem traveling in the far field is large compared to λ, the reactive three-shock theory can be expected to hold (i.e., an equilibrium limit exits). However, no quantitative definition about how and when the two limits apply have been given. Actually, the frozen limit for a detonation is an incomplete concept. The mechanism of how the chemical release breaks down the frozen limit remains unclear. Based on the assumptions of the above two limiting regimes for detonations, Fontin et al. [12] and Li et al. [13] studied the Mach reflection of gaseous detonations by means of experiments and numerical simulations, respectively. Their results suggested that the existence of characteristic length scales (cell size λ in [12] and Zel’dovich–von Neumann–Döring (ZND) detonation thickness Δ in [13]) renders the overall self-similarity of the Mach reflection invalid. In the far field, when λ or Δ are negligible compared to the distance traveled by the Mach stem, the Mach reflection process approaches self-similarity asymptotically, and the local asymptotic trajectory angle exhibits good agreement with the self-similar reactive three-shock theory, indicating that the equilibrium limit is obtained. Note that neither the non-reactive three-shock theory for shock nor the reactive three-shock theory for a detonation discontinuity under the assumption of similarity are able to predict the overall behavior of the Mach reflection of detonation waves. However, within the limit (frozen or equilibrium), the above two three-shock theories can also be applied to describe the local self-similar Mach reflection process either in the near field or in the far field. In their papers, the triple-point trajectory of a detonation Mach reflection appears to maintain self-similarity under the frozen limit (non-reactive three-shock theory) for a relatively large distance (several λ in [12] or 30–40 Δ in [13]). However, this violates the definition of the frozen limit, which requires that the Mach stem travel distance is sufficiently small compared to the characteristic length scales, so as to ensure the detonation front is “frozen”. However, no reason has been given to interpret this discrepancy. In addition, Fontin et al. [12] and Li et al. [13] did not pay additional attention to the details of the transit physics of a ZND detonation in the very near field where the frozen limit may hold.

The transition of the Mach reflection of a ZND detonation from “inert” to “reactive” has been addressed by Ziegler [19] and Radulescu et al. [20] in a small length scale less than a cell size. In Ziegler’s thesis [19], a reduced propane mechanism was used to simulate a double Mach reflection in a direct numerical simulation (DNS) fashion to resolve all the diffusive scales. Actually, he focused on resolving the Mach reflection structures in both viscous and inviscid cases, and the developing process of cellular structures. His work aimed to understand the mechanism of triple-point bifurcation of real detonation fronts by studying the double Mach reflection process. This strategy is in accord with the work of Radulescu et al. [21]. The two studies mentioned above did show some detailed structures of Mach reflection but almost no effort was made to study the frozen limit in respect of the self-similarity problem. The motivation of the present study on Mach reflection is not to reveal the combustion mechanism of a triple-point wave complex in a cellular detonation wave in a small length scale less than a cell size. Instead, we try to study the global front structure of the detonation when encountering a boundary such as the wedge. Thus, a long length scale compared to the cell size is used in the present work. Ziegler [19] and Radulescu et al. [20] considered another pathway to study detonation by focusing on the small scale inside a cell, but this is not the focus of our research.

**Figure 1:**
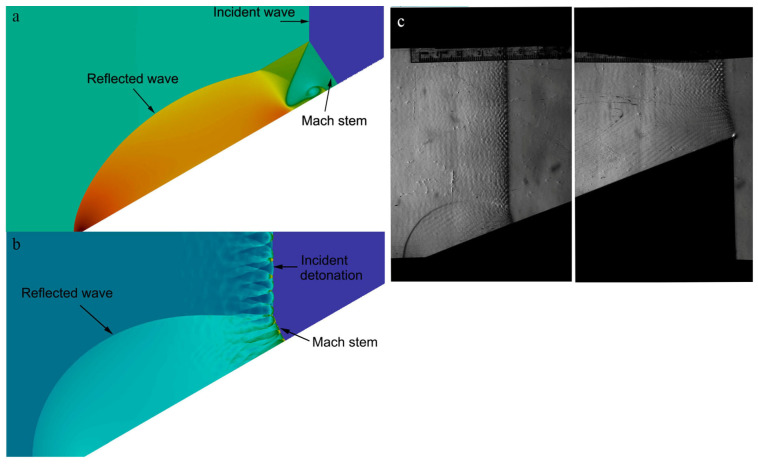
Mach reflection on the wedge: (**a**) inert shock (numerical simulation), (**b**) cellular detonation (numerical simulation), (**c**) cellular detonation (experiments).

Numerical and experimental observations indicate that the detonation front always exhibits cellular properties [12,22,23,24,25]. For a stable detonation, one can locate the triple-point trajectory by observing the shape and size of detonation cells behind the incident front and Mach stem, respectively. However, for an unstable mixture, the triple-point trajectory of Mach reflection is not well-defined due to the cellular properties of the detonation front. Distinguishing the triple-point trajectory from smoke foils, or even from Schlieren photographs, becomes extremely difficult, see Figure 1. One may find that a “smeared out” region [12] rather than a “trajectory” is an acceptable way to describe the boundary. Thus, in the near field where the order of cellular instabilities is comparable with the height of the Mach stem, the triple-point trajectory of Mach reflection with fluctuations becomes ill-defined [26].

All detonation waves are three-dimensional, and the cellular structures may blur the triple-point trajectory throughout the propagation. Thus, it is almost impossible to compare them with an inert shock (or a frozen detonation) without cellular structures in order to elucidate when and where the frozen limit may hold. To avoid the influence of cellular instabilities, numerical simulations of the Mach reflections of ZND detonations as well as an inert shock with the same strength (which is essentially a “frozen” detonation) are both conducted in the present study. We then attempt to perform an accurate comparison between the Mach reflection of a ZND detonation with the self-similar shock case to study the self-similarity problem associated with the onset of the Mach reflection in detail, especially the range of the frozen limit, by focusing on a relatively smaller scale near the wedge apex. Although we calculate the simplest cases of the Mach reflection with planar ZND detonations under the frozen limit in the present study, interesting results are still obtained and much of the physics needed to understand the complex Mach reflection of detonations is also introduced.

## 2. Problem Formulation and Numerical Details

The present simulation of the Mach reflection of ZND detonations is based on the inviscid, reactive Euler equations for two-dimensional flows. Written in conserved form, these equations are represented by:

(1) $$\begin{array}{c} \frac{\partial \rho }{\partial t}+\frac{\partial (\rho u)}{\partial x}+\frac{\partial (\rho v)}{\partial y}=0 \end{array}$$

(2) $$\begin{array}{c} \frac{\partial (\rho u)}{\partial t}+\frac{\partial (\rho u^{2}+p)}{\partial x}+\frac{\partial (\rho uv)}{\partial y}=0 \end{array}$$

(3) $$\begin{array}{c} \frac{\partial (\rho v)}{\partial t}+\frac{\partial (\rho uv)}{\partial x}+\frac{\partial (\rho v^{2}+p)}{\partial y}=0 \end{array}$$

(4) $$\begin{array}{c} \frac{\partial E}{\partial t}+\frac{\partial ((E+p)u)}{\partial x}+\frac{\partial ((E+p)v)}{\partial y}=0 \end{array}$$

(5) $$\begin{array}{c} \frac{\partial (\rho y_{I})}{\partial t}+\frac{\partial (\rho uy_{I})}{\partial x}+\frac{\partial (\rho vy_{I})}{\partial y}=\omega_{I} \end{array}$$

(6) $$\begin{array}{c} \frac{\partial (\rho y_{R})}{\partial t}+\frac{\partial (\rho uy_{R})}{\partial x}+\frac{\partial (\rho vy_{R})}{\partial y}=\omega_{R} \end{array}$$

assuming a polytropic equation of state and an ideal thermal equation of state:

(7) $$\begin{array}{c} E=\frac{p}{\gamma -1}+\frac{\rho (u^{2}+v^{2})}{2}-\rho y_{R}Q \end{array}$$

(8) $$\begin{array}{c} p=\rho RT \end{array}$$

The conservation laws are coupled with a two-step chain-branching-type reaction model [16,17]. The first step represents a thermally neutral induction zone, with a temperature-sensitive Arrhenius form of the reaction rate given by:

(9) $$\omega_{I}=H\left(1-y_{I}\right)\rho k_{I}\exp\left[E_{I}\left(\frac{1}{RT_{S}}-\frac{1}{RT}\right)\right]$$

where $H(1-y_{I})$ is a step function defined as

(10) $$H\left(1-y_{I}\right)=\left\{\begin{array}{c} 1\;\;\;\;\mathrm{if}\;\;y_{I}<1\\ 0\;\;\;\;\mathrm{if}\;\;y_{I}\geq 1 \end{array}\right.$$

At the end of the induction zone, the second step describes the rapid energy release after the branched-chain thermal explosion and the slow heat release in the radical recombination stage. The reaction rate for this step is given by:

(11) $$\omega_{R}=\left(1-H\left(1-y_{I}\right)\right)\rho k_{R}\left(1-y_{R}\right)\exp\left(\frac{E_{R}}{RT}\right)$$

The induction length $\Delta_{I}$ is controlled by the induction rate constant $k_{I}$, and the reaction rate constant $k_{R}$ determines the length of the reaction zone $\Delta_{R}$. Thus, the reaction rate constant $k_{R}$ is also used as a bifurcation parameter to control the ratio of the reaction length $\Delta_{R}$ to the induction length $\Delta_{I}$, or the stability of a detonation wave. Generally, the induction length is simply defined as the length of the thermally neutral period. As pointed out by Ng et al. [27], no standard definition exits for the reaction length $\Delta_{R}$. In the ZND detonation model, we determine this scale from the thermicity profile, i.e., $\Delta_{R}$ is the half-height width of the thermicity pulse. Thus, the overall thicknesses of the detonation front can be obtained, i.e., $\Delta =\Delta_{I}+\Delta_{R}$.

In the present calculation, the global heat release *Q* is determined in order to reproduce the correct CJ Mach number, given for a perfect gas [28] by:

(12) $$\frac{Q}{RT_{0}}=\frac{\gamma }{2(\gamma^{2}-1)}{\left(M_{\mathrm{CJ}}-\frac{1}{M_{\mathrm{CJ}}}\right)}^{2}$$

Some reaction parameters are evaluated and shown in Table 1. Typical values for $\frac{E_{I}}{RT_{\mathrm{VN}}}$ usually range from 4 (for hydrogen–oxygen mixtures) to 12 (for heavy hydrocarbon mixtures) [17]. In contrast, the second step involves only reactions between energetic free radicals. For typical chain-branching reactions, the induction stage generally has a larger activation energy as compared to the reaction stage. Hence, we simply set $\frac{E_{R}}{RT_{\mathrm{VN}}}=1$ for the present study. The simplest way to obtain an inert shock within the framework of ZND detonations with the present two-step model is to reduce the induction rate constant $k_{I}$ to obtain a very large induction zone. The induction zone is thermally neutral, and in essence, the “frozen” ZND detonation corresponds to an inert shock. Thus, the isentropic exponent and the Mach number of inert shock waves are the same as ZND detonation waves. By maintaining $k_{I}$ as a constant value and varying $k_{R}$, we obtained ZND detonations with the same induction lengths but different reaction widths, i.e., Case-A–Case-F in Table 2. The effect of activation energies was also studied by additionally including Case-G and Case-H in Table 2 for comparison purposes. The two-step model, as mentioned by Short and Sharpe [16], serving as the simplest chain-branching kinetics, can mimic some features of the chain-branching chemistry. Of course, the two-step model cannot describe the physics in a quantitative manner compared to the detailed chemical kinetics. However, the two-step model does have its unique features, such as decoupled detonation parameters rendering a systematical parameter study, which is not possible when using the detailed chemical kinetics.

**Figure 2:**
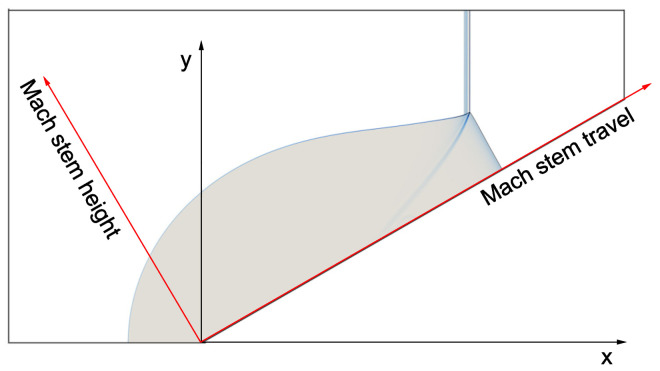
Setup of the computational domain.

Adaptive mesh refinement techniques are widely used in computational mechanics to increase the computation efficiency. The pseudo-arc-length method for hyperbolic conservation laws has been successfully used in the simulation of shock and detonation waves [29,30] by twisting and converging the grid near a discontinuity. Structured adaptive mesh refinement techniques that locally split the grid are also popular in simulation problems with large discontinuities. In the present study, the governing equations were solved using a parallel AMROC code [31] built with a block-structured adaptive mesh refinement (AMR) technique [32]. A fractional step method was used to decouple the hydrodynamic transport and the chemical reaction numerically. The reactive Euler equations were then solved with an explicit second-order Godunov-type scheme incorporating a hybrid Roe-solver-based method. Slip wall conditions were imposed on solid boundaries. The upper and lower boundaries as well as the wedge surface were all set as solid walls. The outlet boundary condition was imposed on the right boundary. The simulation was initialized with a planar ZND detonation placed at the wedge apex. The left boundary was fixed with the Chapman–Jouguet (CJ) solution such that there was no expansion wave behind the CJ point of the ZND detonation wave. The computational domain with a $30^{\circ }$ wedge is shown in Figure 2. The default wedge length was set to 400$\Delta_{I}$ throughout the present study except in Section 3.1, where a wedge of length of 800$\Delta_{I}$ was used to compare the Mach reflection of a ZND detonation and a cellular detonation. Besides the original coordinates (x–y), a convenient coordinate system (Mach stem travel–Mach stem height) was also used to facilitate the study to the self-similarity of Mach reflection. In the present work, the wedge boundary was implemented using the embedded boundary condition, which has been well described and verified in [31]. This permitted the use of a Cartesian grid upon which the boundary geometry was mapped.

**Figure 3:**
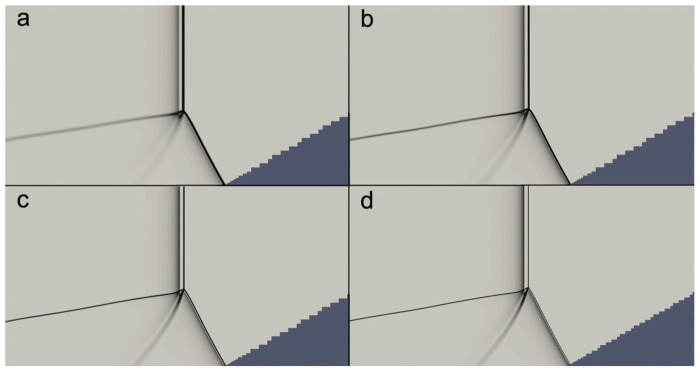
Schlieren plots of flow field near the triple-point for different grid resolutions: (**a**) 8 pts/$\Delta_{I}$, (**b**) 16 pts/$\Delta_{I}$, (**c**) 32 pts/$\Delta_{I}$, (**d**) 64 pts/$\Delta_{I}$.

**Figure 4:**
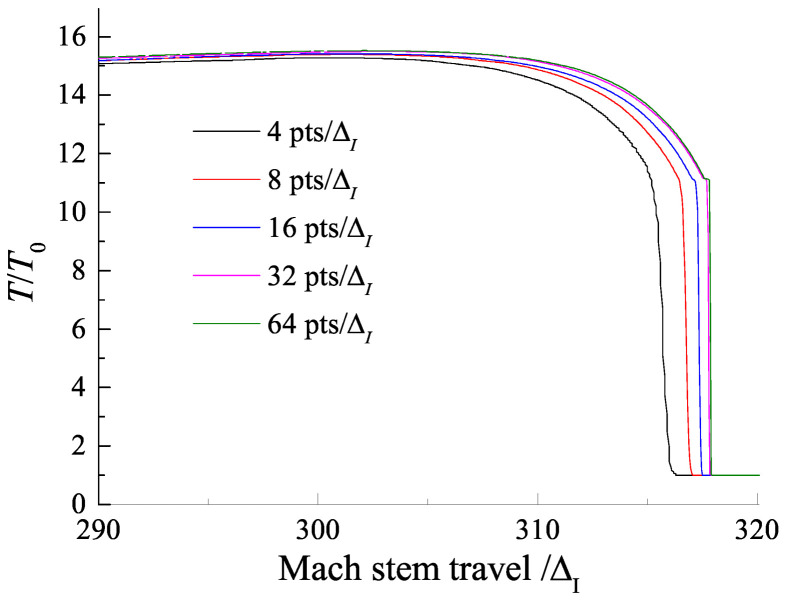
Temperature profiles along the wedge surface for different grid resolutions.

The convergence of the numerical results in the present study was tested by considering different grid resolutions for the Mach reflection of a ZND detonation on a wedge of $30^{\circ }$ with Case-A. Figure 3 shows the density gradient of the Mach reflection at the same position for different grid resolutions. It is observed that there is a convergence of the features of the flow field as the resolution increases. Similar grid resolution convergence can also be seen in Figure 4, which shows the temperature profiles behind the Mach stem front along the wedge surface corresponding to the case in Figure 3. For a grid resolution of 32 pts/$\Delta_{I}$, the temperature profile is in good agreement with that of the highest grid resolution of 64 pts/$\Delta_{I}$. Hence, in the present work, a maximum grid resolution of 32 pts/$\Delta_{I}$ with four levels of Cartesian mesh adaptation with refinement factors (2, 2, 2, 4) was used to ensure that the detailed features of ZND detonation waves were properly resolved.

## 3. Results and Discussion

### 3.1. The Overall Behavior of the Mach Reflection of Detonations on a Wedge

**Figure 5:**
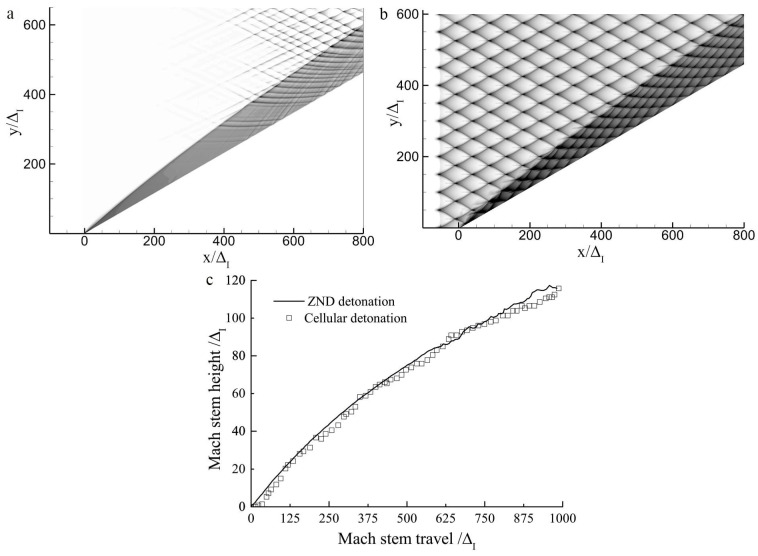
Numerical smoke foils and triple-point trajectories for Case-A with $\theta_{w}=30^{\circ }$: (**a**) Zel’dovich–von Neumann–Döring (ZND) detonation, (**b**) cellular detonation, (**c**) triple-point trajectories.

A two-dimensional planar ZND detonation cannot be maintained because the cellular structures always develop at some distance downstream. Thus, the initial Mach reflection process corresponds to that of a planar ZND detonation as shown in Figure 5a. Then, the weak cellular structures appear on the detonation front such that the Mach reflection of a weak cellular detonation is obtained. Note that it usually takes a long time for a stable planar ZND detonation to evolve into a fully established cellular detonation. In Figure 5b, an initially fully developed cellular detonation with a regular cell pattern is placed immediately before the wedge tip such that this refers to the Mach reflection of a cellular detonation. A comparison of the triple-point trajectories for these two cases is shown in Figure 5c. As can be observed, the triple-point trajectories for the two cases almost coincide except for some fluctuations due to cellular instability, thereby indicating that the Mach reflection of a cellular detonation essentially behaves like that of a ZND detonation wave. The comparison suggests that even though the Mach reflection of a ZND detonation never occurs in reality, it still provides an alternative, simple, and meaningful way to interpret the physics of the Mach reflection of cellular detonations. Note that the limitation of using a ZND detonation is that the physics may not be “real” and a characteristic length scale, i.e., the cell size, is also ignored. By using a ZND detonation, on the one hand, we do lose some intrinsic information about the real physics, but on the other hand, the self-similarity that can be clearly observed renders an accurate comparison with inert shock possible. Thus, in the following study, only the stage before the presentation of cellular instabilities (i.e., a wedge length of 400 $\Delta_{I}$) is investigated, as shown in Figure 5a, in order to ensure the Mach reflection of a ZND detonation other than a cellular detonation. Case-A was chosen as a default case in the present study for simulation unless specified.

### 3.2. Evolution of the Mach Reflection Configuration

#### 3.2.1. $L<5\Delta_{I}$

**Figure 6:**
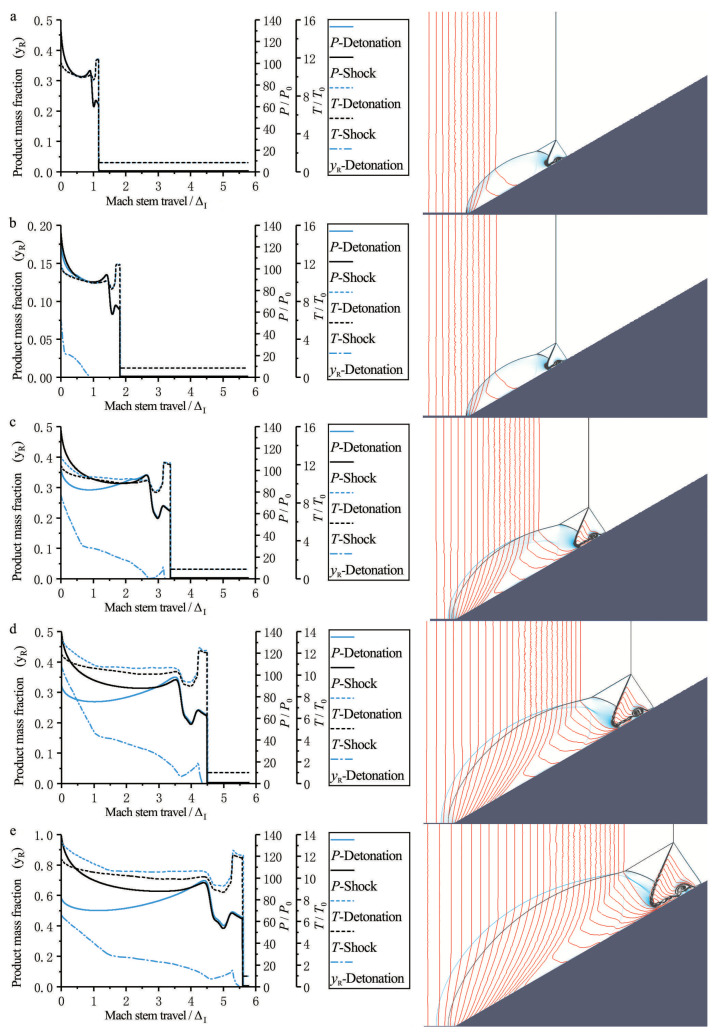
Sequences of combined contour plots for the Mach reflection of a ZND detonation (Case-A) and an inert shock with $\theta_{w}=30^{\circ }$ at different positions: (**a**) 1$\Delta_{I}$, (**b**) 2$\Delta_{I}$, (**c**) 3$\Delta_{I}$, (**d**) 4$\Delta_{I}$, (**e**) 5$\Delta_{I}$. (The red lines represent the chemical reaction progress).

The Mach reflection of an inert shock is self-similar because of the absence of characteristic length scales. In a ZND model, the detonation front can be treated as a neutral shock front followed by a region in which the chemical energy is released. Thus, when a ZND detonation arrives at the wedge tip, a Mach reflection of the neutral shock front is first observed, as shown in Figure 6a. This reflection corresponds to the so-called frozen limit. Note that this can only occur within an extremely small scale, i.e., of the order of the induction length $\Delta_{I}$, when the Mach stem is truly “frozen”. Obviously, in this region, the Mach reflection is exactly self-similar, and the triple-point trajectory is a straight line. In Figure 6a, it is also observed that the pressure and temperature profiles in the case of a ZND detonation coincide with those for an inert shock with the same strength. However, as the ZND detonation propagates forwards, the following reaction front also reflects on the wedge, and the chemical release begins to influence the Mach reflection configuration. As shown in Figure 6a–e, the pressure of the reaction zone behind the leading front decreases because of the chemical release in the reaction zone, resulting in a bowed reflected wave that travels slightly faster than in the case of an inert shock. Thus, at this very moment, the Mach reflection configuration in the case of a ZND detonation does not coincide exactly with that of an inert shock with the same strength, except in respect of the leading front, which has not been disturbed yet. Thus, the triple-point trajectory of the Mach reflection in the ZND model is still a straight line, and coincides with that in the case of an inert shock.

An interesting phenomenon occurs during the onset of the Mach reflection of a ZND detonation (i.e., the transition of the Mach stem from “frozen” to “reactive”), as shown in Figure 6. A chemical reaction zone induced by the reflection follows behind the inert Mach stem, with an induction length approximately equal to that behind the incident detonation wave (see Figure 6b). Then, another reactive front appears immediately behind the inert Mach stem due to the shock ignition, as shown in Figure 6c. Thus, two chemical reaction zones both exist at this very moment, one from the reflection of the original ZND detonation and the other due to the leading shock ignition. An unreacted “gap” that has already been compressed (or shocked) by the Mach stem front is now located between these two reaction zones. This unreacted “gap” is in accordance with the so-called “hot spot” as described by Zel’dovich et al. [33] and many others [34,35,36]. In Figure 6d,e, the gap between the two chemical reaction zones emerges, and then a continuous reaction zone is finally created behind the Mach stem. The emergence of the “hot spot” causes compression waves that develop subsequently to catch up with the precursor Mach stem front ahead of it, and forms a more overdriven detonation. Finally, a typical Mach reflection configuration for a ZND detonation is fully established, with a reactive Mach stem (overdriven detonation) of shorter induction length than that of the incident detonation (i.e., a CJ detonation).

#### 3.2.2. $5\Delta_{I}<L<40\Delta_{I}$

**Figure 7:**
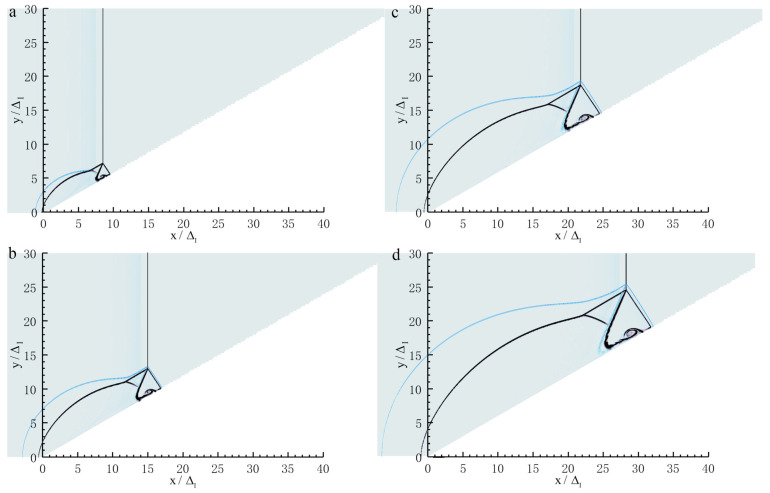
Schelieren photography of Mach reflection of a ZND detonation for Case-A with $\theta_{w}=30^{\circ }$ at different positions: (**a**) 8$\Delta_{I}$, (**b**) 15$\Delta_{I}$, (**c**) 22$\Delta_{I}$, (**d**) 28$\Delta_{I}$. (blue line—ZND detonation, black line—inert shock).

**Figure 8:**
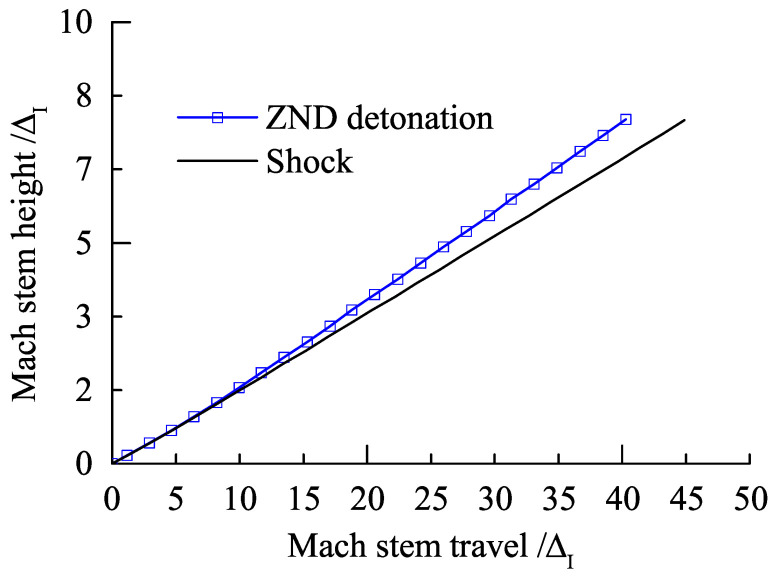
Comparison of the triple-point trajectories of a ZND detonation and an inert shock in the very near field for Case-A with $\theta_{w}=30^{\circ }$.

**Figure 9:**
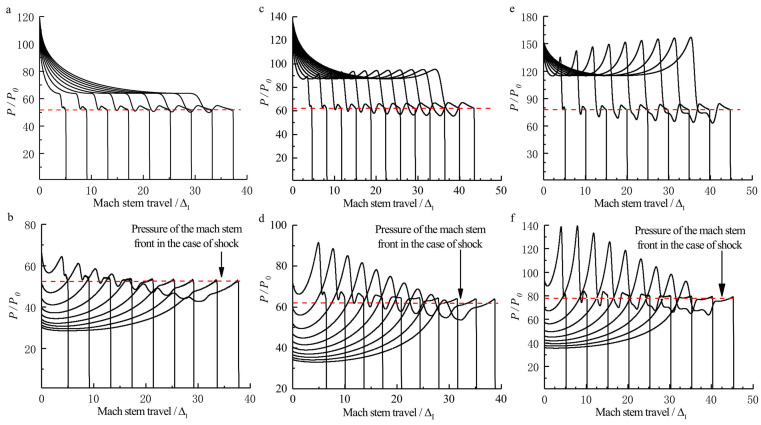
Pressure profiles at different times of the Mach stem front along the wedge surface within $5\Delta_{I}<L<40\Delta_{I}$: (**a**) inert shock, $\theta_{w}=20^{\circ }$, (**b**) ZND detonation, $\theta_{w}=20^{\circ }$, (**c**) inert shock, $\theta_{w}=30^{\circ }$, (**d**) ZND detonation, $\theta_{w}=30^{\circ }$, (**e**) inert shock, $\theta_{w}=40^{\circ }$, (**f**) ZND detonation, $\theta_{w}=40^{\circ }$.

Figure 7 shows a sequence of numerical Schlieren photography of the Mach reflection of a ZND detonation ($5\Delta_{I}<L<40\Delta_{I}$). Note that the Mach reflection of an inert shock with the same strength as the ZND detonation is also plotted in Figure 7 for comparison. In Figure 7a, the coincidence of Mach reflection in the vicinity of the triple-point in both of the two cases is observed. However, the bowed reflected shocks separate as a result of the pressure decay in the chemical reaction zone in the case of a ZND detonation, whereas in Figure 7b–d, the Mach reflection of the ZND detonation is no longer in accordance with that in the case of an inert shock except for the incident waves, which have the same strength. It is well known that the Mach reflection of an inert shock is self-similar by ignoring the viscosity and dissociation effect, indicating a straight triple-point trajectory from the wedge tip. Figure 8 shows that, within a distance of approximately 10 $\Delta_{I}$, the triple-point trajectory of the Mach reflection of a ZND detonation is the same as that of an inert shock. Subsequently, the triple-point trajectory of the Mach reflection of a ZND detonation deviates from the triple-point trajectory of the Mach reflection of an inert shock and rises, thereby suggesting that the strength (or the velocity) of the Mach stem increases. This is probably due to the compression waves created by the hot spot during the transition to the Mach reflection of a ZND detonation (or the formation of the reactive Mach stem) near the wedge tip as discussed above. As shown in Figure 9, the pressure of the Mach stem of an inert shock remains constant due to self-similarity. However, the Mach stem pressure in the case of a ZND detonation is approximately 5% higher than that in the case of an inert shock, due to the “hot spot” effect discussed above, indicating the loss of self-similarity. Thus, if we only consider a straight triple-point trajectory to characterize self-similarity, the frozen limit is about 10$\Delta_{I}$ (i.e., of the order of a Δ).

#### 3.2.3. $40\Delta_{I}<L<400\Delta_{I}$

**Figure 10:**
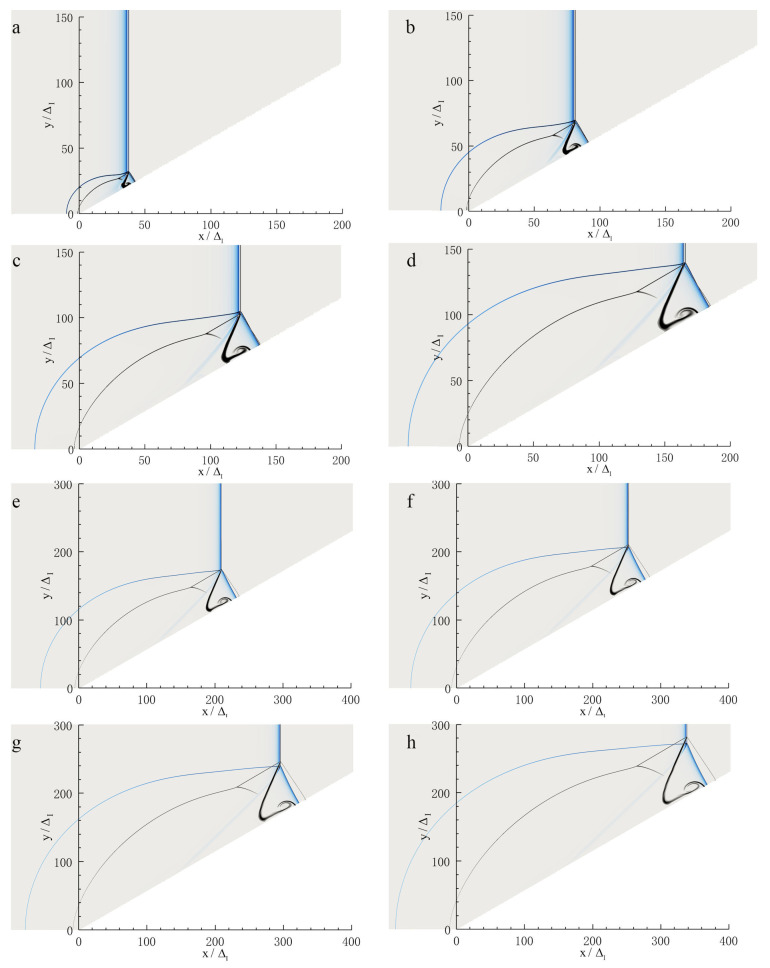
Schlieren photography of Mach reflection of a ZND detonation for Case-A with $\theta_{w}=30^{\circ }$ at different positions: (**a**) 36$\Delta_{I}$, (**b**) 80$\Delta_{I}$, (**c**) 120$\Delta_{I}$, (**d**) 165$\Delta_{I}$, (**e**) 200$\Delta_{I}$, (**f**) 250$\Delta_{I}$, (**g**) 280$\Delta_{I}$, (**h**) 330$\Delta_{I}$.

**Figure 11:**
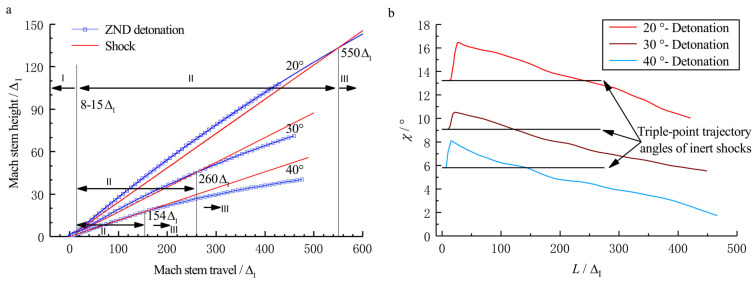
Comparison of (**a**) the triple-point trajectories and (**b**) their slopes of ZND detonations (Case-A) and inert shocks with $\theta_{w}=20^{\circ },30^{\circ },40^{\circ }$ ($40\Delta_{I}<L<400\Delta_{I}$).

**Figure 12:**
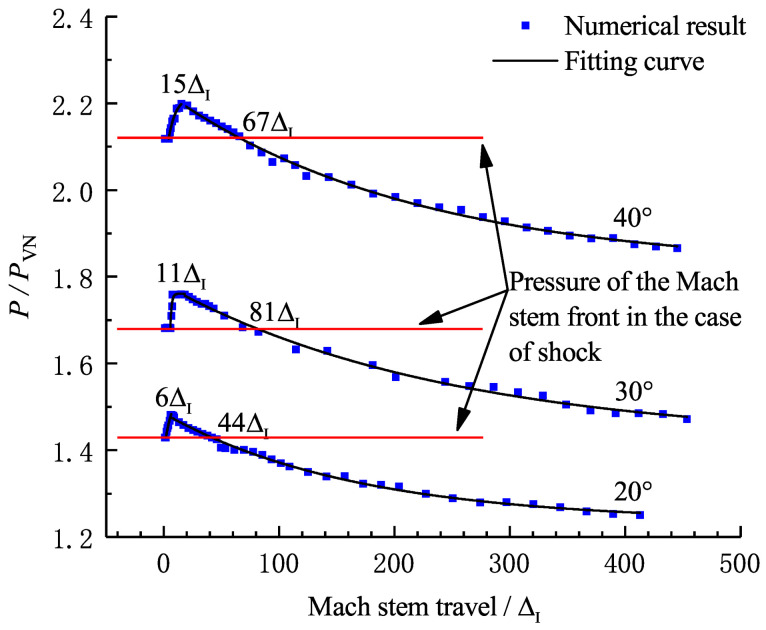
Pressure history of the Mach stem front of a ZND detonation and an inert shock for Case-A with $\theta_{w}=20^{\circ },30^{\circ },40^{\circ }$ ($40\Delta_{I}<L<400\Delta_{I}$).

In this region ($40\Delta_{I}<L<400\Delta_{I}$), the Mach reflection of a ZND detonation continues to be unsteady as shown in Figure 10. The triple-point in the case of a ZND detonation continues to remain above that of an inert shock, yet as the detonation front travels forwards, the triple-point in the case of a ZND detonation starts to fall and coincides with the triple-point in the case of an inert shock at the moment of Figure 10f. The triple-point trajectories and their slopes in the case of a ZND detonation are shown in Figure 11. The corresponding pressure curves of the Mach stem are shown in Figure 12. The pressure increase results in the triple-point trajectory rising, whereas a decrease causes the triple-point trajectory to decay. This is in accordance with the analysis of Ben-Dor [4], in which the effect of shock strength on the triple-point trajectory angle of Mach reflection, i.e., the triple-point trajectory angle increase as the shock strength increases, was investigated. The increase in the Mach stem pressure is due to the “hot spot” effect discussed above, whereas the subsequent decrease is because of the effect of length scales on the Mach reflection configuration, which has been fully discussed by Fortin et al. [12] and Li et al. [13].

### 3.3. Length Scale Effect

**Figure 13:**
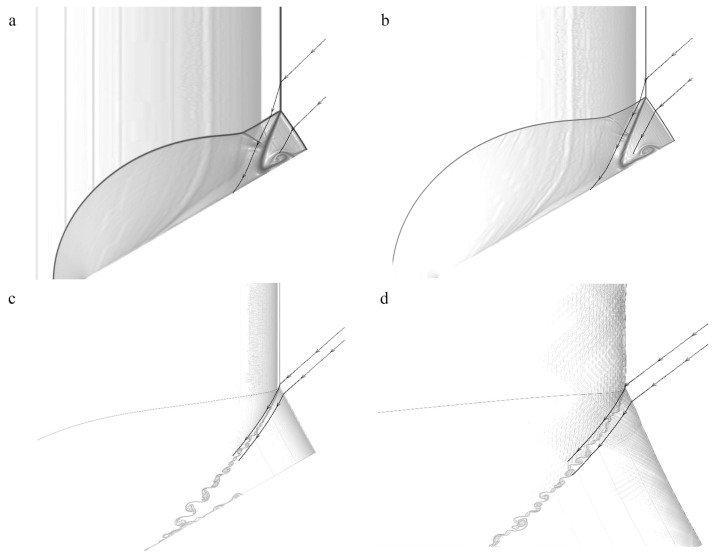
Streamlines of Mach stem in a frame fixed at the triple-point for Case-A with $\theta_{w}=30^{\circ }$ at different positions: (**a**) 10$\Delta_{I}$, (**b**) 20$\Delta_{I}$, (**c**) 160$\Delta_{I}$, (**d**) 400$\Delta_{I}$.

The triple-point trajectory can be divided into three parts (I, II, and III) as shown in Figure 11. In part I, the Mach stem can be regarded as a “frozen” detonation, the reactive front of which is far behind the precursor shock front compared to the CJ detonation. Thus, the triple-point trajectories for the two examined cases (inert shock and ZND detonation) coincide. In part II, during the transition of the Mach stem from “frozen” to “reactive”, the temperature and pressure both increase, and the propagation velocity accelerates due to the “hot spot” effect. This causes the triple-point trajectory to rise from the inert shock case. In part III, the reactive Mach stem behaves as an overdriven detonation, which can be influenced by the flow field behind the Mach stem front. The bowed reflected wave of the Mach reflection can be regarded as the rear boundary of the reactive Mach stem. Furthermore, the pressure decrease caused by the chemical energy release in the reaction zone attenuates the reactive Mach stem. The pressure changes are in accordance with the rise and fall of the triple-point trajectory of the Mach reflection of ZND detonations. A pressure increase corresponds to a rise in the triple-point trajectory, whereas a pressure decrease causes the triple-point trajectory to decay. Figure 13 shows the streamlines of Mach reflection in the case where $\theta_{w}=30^{\circ }$ at different positions in a frame fixed at the triple-point. The deflection of the flow across the incident wave, reflected wave, and Mach stem can be clearly observed by following the streamline. In the near field with short travel of the Mach stem, the streamlines in the vicinity of the triple-point are straight lines unless passing through a discontinuity due to the domination of freeze of chemical reaction. However, as the Mach stem travel becomes larger, the exothermic process will become more significant and the flow will deflect back to the direction of the inflow ahead of the reflected shock or Mach stem within the reaction zone. The decreasing deflection angle θ therefore results in the decrease of the triple-point trajectory angle as shown in Figure 11. Conclusively, the progress of the exothermic process controls the change of the triple-point trajectory and its slope.

**Figure 14:**
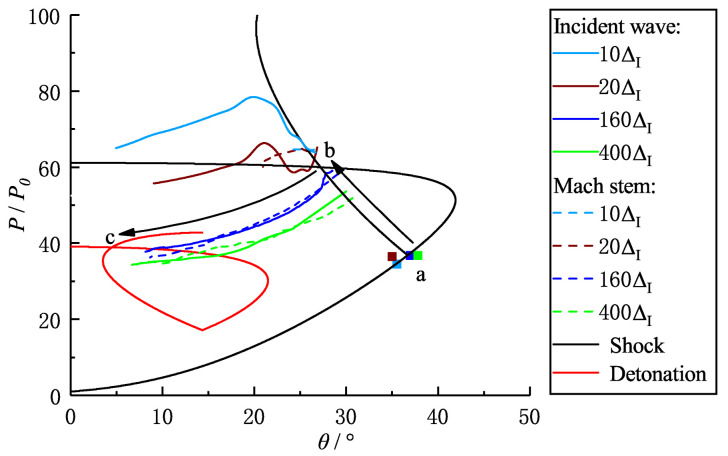
Shock and detonation polars as well as the numerical results for Case-A with $\theta_{w}=30^{\circ }$.

The shock and detonation polars are extensively used in the Mach and regular reflection fields to predict the flow states and the critical angle. Note that the shock or detonation polars are obtained from the inert or reaction three-shock theory based on the assumption of shock or detonation discontinuity. Thus the shock and detonation polars can only be used to predict the equilibrium states, and are not capable of describing the transient processes. Figure 14 shows the shock and detonation polars based on the three-shock theory and the numerical results extracted from the streamlines, as shown in Figure 13. According to the inert shock polar, points a and b at the shock polars refer to the flow states (pressure and flow deflection angle θ) behind the incident wave and reflected wave (Mach stem), respectively. Point c at the detonation polars corresponds to flow states behind the reflected wave or reactive Mach stem based on the reactive three-shock theory. It can be observed that, as the detonation travels along the wedge in the far-field, the streamlines approach connecting points b and c, indicating the transient reaction process from the frozen to equilibrium states. It also suggests that the flow deflection angle θ approaches point c in which the state is obtained based on the assumption of detonation discontinuity (reactive three-shock theory).

**Figure 15:**
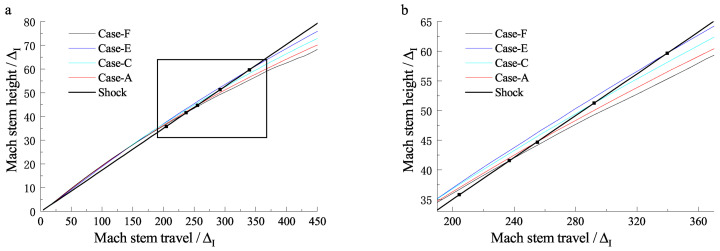
Triple-point trajectories for different cases: (**a**) original figure, (**b**) local enlargement.

**Figure 16:**
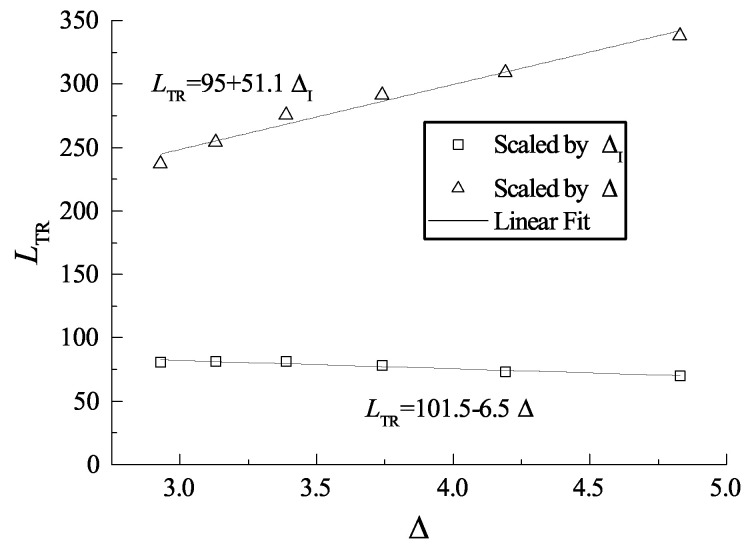
Correlation of the transition length with Δ.

**Figure 17:**
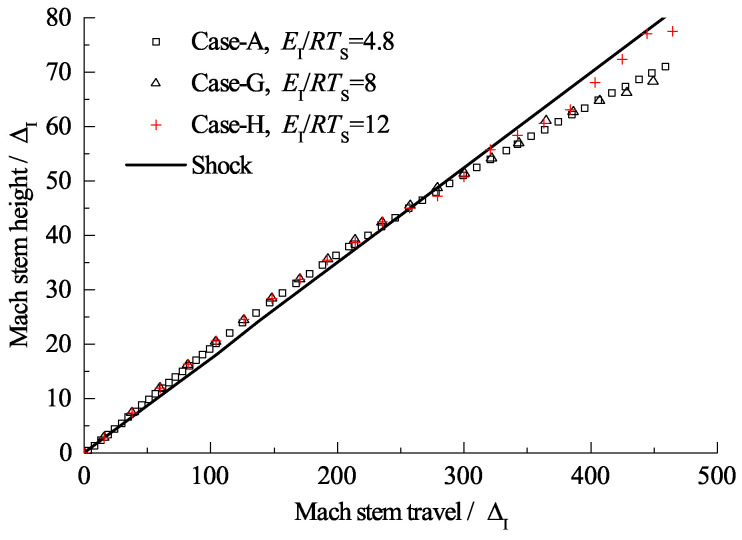
Triple-point trajectories for cases with different activation energies.

By maintaining the induction length at a constant value and varying the reaction zone length, ZND detonations with different Δ are obtained. Figure 15a shows the triple-point trajectories for different cases, and the curves within the black square are enlarged and shown in Figure 15b. Here, we define the Mach stem travel from the wedge apex to the point where the triple-point trajectory reunites with the shock curve (illustrated as the black points in Figure 15) as the transition length. It is observed that Δ can influence the distance for which the frozen limit holds. Figure 16 shows the relationship of the scaled transition length with detonation thickness Δ. For the cases studied in this section, the transition length is approximately 50–60 Δ and has a linear correlation with Δ. Note that the correlation may vary due to a different definition of the reaction length. As discussed earlier, the cellular structures of unstable mixtures develop faster than those of stable ones and can result in a more fluctuated triple-point trajectory. Figure 17 shows the triple-point trajectories for Case-A, Case-G, and Case-H, which have different activation energies. The three triple-point trajectories coincide before cellular stabilities appear. This confirms that it is the length scale effect, rather than the activation energy, that dominates the Mach reflection of detonations. However, the activation energy does influence the fluctuation of the triple-point trajectory via changing cellular stabilities.

## 4. Conclusions

The present study involved a numerical investigation of the problem of Mach reflection of ZND detonations to investigate qualitatively the onset of the Mach reflection in the near field. A simulation of the self-similar Mach reflection of an inert shock wave was also conducted for reference purposes. The obtained results indicate that the assumption of a self-similar Mach reflection for a ZND detonation is only valid (i.e., the self-similar three-shock theory applies) for an extremely small distance along the wedge surface, which is consistent with the argument presented by Akbar [11]. Afterwards, the reactive Mach stem is found to accelerate rapidly to a more overdriven detonation, which subsequently decays due to the effect of chemical release and length scale. The determined acceleration is most likely due to the influence of the compression waves generated by the “hot spot” behind the Mach stem, which appears when the reactive Mach stem develops. It should be noted here that, even though the frozen limit is valid only for a very short distance of the Mach stem travel when the flow is really “frozen” (without reaction), the triple-point trajectory was found to be close to the self-similar results for an inert shock when the flow was no longer “frozen”. Thus, from an engineering perspective, the self-similar three-shock theory can be used to obtain approximate predictions of the triple-point trajectory for a much longer Mach stem travel distance than that considered in the present paper. This finding can also explain why the triple-point trajectories of reactive Mach reflections obtained in many experiments conform to the predictions of the self-similar non-reactive three-shock theory over quite a large Mach stem travel distance.

It should be pointed out that the conclusion obtained in the present study may differ from that of the experiments due to the following aspects. Firstly, the Mach reflection of a ZND detonation ignores the cellular stability effect. Secondly, although the two-step model used in the present study is far from real chemistry, it is able to qualitatively represent some hydrodynamic features of detonations. Thirdly, the viscous effect was not considered in the present study. However, it is questionable as to whether or not the viscous effect would be able to influence the Mach reflection process, and more work is needed to clarify this problem.

## Figures and Tables

**Table 1:** Length scale parameters.

	$\frac{E_{I}}{{RT}_{S}}$	$\frac{E_{R}}{{RT}_{S}}$	$k_{I}$	$k_{R}$	$\Delta_{I}$	$\Delta_{R}$	Δ
			($m^{3}$/kg/s)	($m^{3}$/kg/s)	(mm)	(mm)	(mm)
Case-A	4.8	1.0	$4.229\times 10^{6}$	$0.9\times 6.096\times 10^{6}$	0.1	0.213	0.313
Case-B	4.8	1.0	$4.229\times 10^{6}$	$0.8\times 6.096\times 10^{6}$	0.1	0.239	0.339
Case-C	4.8	1.0	$4.229\times 10^{6}$	$0.7\times 6.096\times 10^{6}$	0.1	0.274	0.374
Case-D	4.8	1.0	$4.229\times 10^{6}$	$0.6\times 6.096\times 10^{6}$	0.1	0.319	0.419
Case-E	4.8	1.0	$4.229\times 10^{6}$	$0.5\times 6.096\times 10^{6}$	0.1	0.383	0.483
Case-F	4.8	1.0	$4.229\times 10^{6}$	$1.1\times 6.096\times 10^{6}$	0.1	0.193	0.293
Case-G	8.0	1.0	$4.229\times 10^{6}$	$0.9\times 6.096\times 10^{6}$	0.1	0.213	0.313
Case-H	12.0	1.0	$4.229\times 10^{6}$	$0.9\times 6.096\times 10^{6}$	0.1	0.213	0.313

**Table 2:** Reaction parameters.

Model Parameters	Value	Unit
*R*	218.79	J/kg·K
$p_{0}$	50	kPa
$T_{0}$	295	K
$\rho_{0}$	0.775	kg/$m^{3}$
$Q/RT_{0}$	19.7	
γ	1.44	
$M_{\mathrm{CJ}}$	5.6	

## Nomenclature

λ	Cell size
$\Delta_{I}$, $\Delta_{R}$, Δ	Induction length, reaction length, detonation thickness
$M_{\mathrm{CJ}}$	Mach number of the Chapman–Jouguet detonation wave
$\theta_{w}$	wedge angle
$p_{0}$, $T_{0}$, $\rho_{0}$	Initial pressure, initial temperature, initial density
$p_{\mathrm{VN}}$, $T_{\mathrm{VN}}$	von Neumann pressure and temperature
$T_{s}$	von Neumann pressure
$k_{I}$, $k_{R}$	Induction and reaction rate constants
$E_{I}$, $E_{R}$	Induction and reaction activation energies
$y_{I}$, $y_{R}$	Induction and reaction progress parameters
*Q*	Heat release
*R*	Gas constant
γ	Specific heat ratio
*L*	Distance of the Mach stem travel
**Subscripts**	
w	wedge
I	Induction
R	Reaction
CJ	Chapman–Jouguet
ZND	Zeldovich–von Neumann–Döring
